# Solution Structure of the Oncogenic MIEN1 Protein Reveals a Thioredoxin-Like Fold with a Redox-Active Motif

**DOI:** 10.1371/journal.pone.0052292

**Published:** 2012-12-20

**Authors:** Chun-Hua Hsu, Tang-Long Shen, Chi-Fon Chang, Yu-Yung Chang, Lin-Ya Huang

**Affiliations:** 1 Department of Agricultural Chemistry, National Taiwan University, Taipei, Taiwan; 2 Degree Program of Genome and Systems Biology, National Taiwan University, Taipei, Taiwan; 3 Department of Plant Pathology and Microbiology, National Taiwan University, Taipei, Taiwan; 4 Genomics Research Center, Academia Sinica, Taipei, Taiwan; George Washington University, United States of America

## Abstract

The novel tumor biomarker *MIEN1*, identified by representational difference analysis, is overexpressed in breast cancer and prostate cancer. MIEN1 is considered an oncogenic protein, because MIEN1 overexpression functionally enhances migration and invasion of tumor cells via modulating the activity of AKT. However, the structure and molecular function of MIEN1 is little understood. Here, we report the solution structure of MIEN1, which adopts a thioredoxin-like fold with a redox-active motif. Comparison of backbone chemical shifts showed that most of the residues for both oxidized and reduced MIEN1 possessed the same backbone conformation, with differences limited to the active motif and regions in proximity. The redox potential of this disulfide bond was measured as −225 mV, which compares well with that of disulfides for other thioredoxin-like proteins. Overall, our results suggest that MIEN1 may have an important regulatory role in phosphorylation of AKT with its redox potential.

## Introduction

Migration and invasion enhancer 1 (*MIEN1*), previously named *C35/C17orf37*, is a novel gene identified by representational difference analysis of breast tumor and normal cells [1]. *MIEN1* locates within the minus strand of the human chromosomal region 17q12-21, a “hot spot” locus of cancer named *HER2* amplicon [2]. *MIEN1* expression is positively associated with grade and stage of breast cancer but has low expression in normal tissue [1]. Moreover, protein expression of MIEN1 was increased in patients with metastasis of breast cancer to lung and liver, which indicates that MIEN1 may be an important mediator of cancer cell metastasis [1], [3]. In contrast, the minimal MIEN1 expression found in 38 different normal tissues examined suggests that MIEN1 is a cancer-specific protein and could be a novel tumor biomarker [1].

MIEN1 gene encodes a 13-kDa protein that does not have sequence similarity with any known proteins [1], [3], [4]. Analysis of its protein sequence revealed that MIEN1 contains a canonical immunoreceptor tyrosine-based activation motif, common in receptors of the immune system [5], which has been associated with cell transformation by activating downstream Syk signaling [6]. As well, MIEN1 protein contains a consensus sequence for prenylation comprising the last 4 amino acids, CVIL, at the C terminus, a CAAX motif predicted to be post-translationally modified by protein geranylgeranyltransferase-1 [7]. Western blot analysis of cellular fractions and immunocytochemical detection by confocal microscopy, as well as total internal reflection fluorescence microscopy, showed endogenous expression of MIEN1 predominantly in the cytosolic cell compartment, with dense spots around the cell membrane [3]. Geranylation of MIEN1 at the CAAX motif facilitated the association of the protein to the inner leaflet of plasma membrane, enhancing the migratory phenotype of cells by inducing increased filopodia formation and potentiating directional migration [8]. MIEN1 protein is a signaling molecule promoting cell migration and invasion through a PI3K/Akt pathway, thereby transcriptionally upregulating the NF-κB downstream target genes (matrix metlloprotease 9, urokinase plasminogen activator, and vascular endothelial growth factor) and thus resulting in cancer metastasis [3]. However, little is known about the structural and functional relationship of MIEN1 to signaling.

Here, we report the solution structure of MIEN1 and elucidate the plausible molecular function of MIEN1. Because the amount of phosphorylated AKT is increased with MIEN1 overexpression and thus enhances cell migration and invasion, we suggest a molecular mechanism of redox-sensitive activation for AKT based on our understanding of the structure and biochemical function of MIEN1.

## Results

### Secondary Structure and Thermal Stability of MIEN1

**Figure 1:**
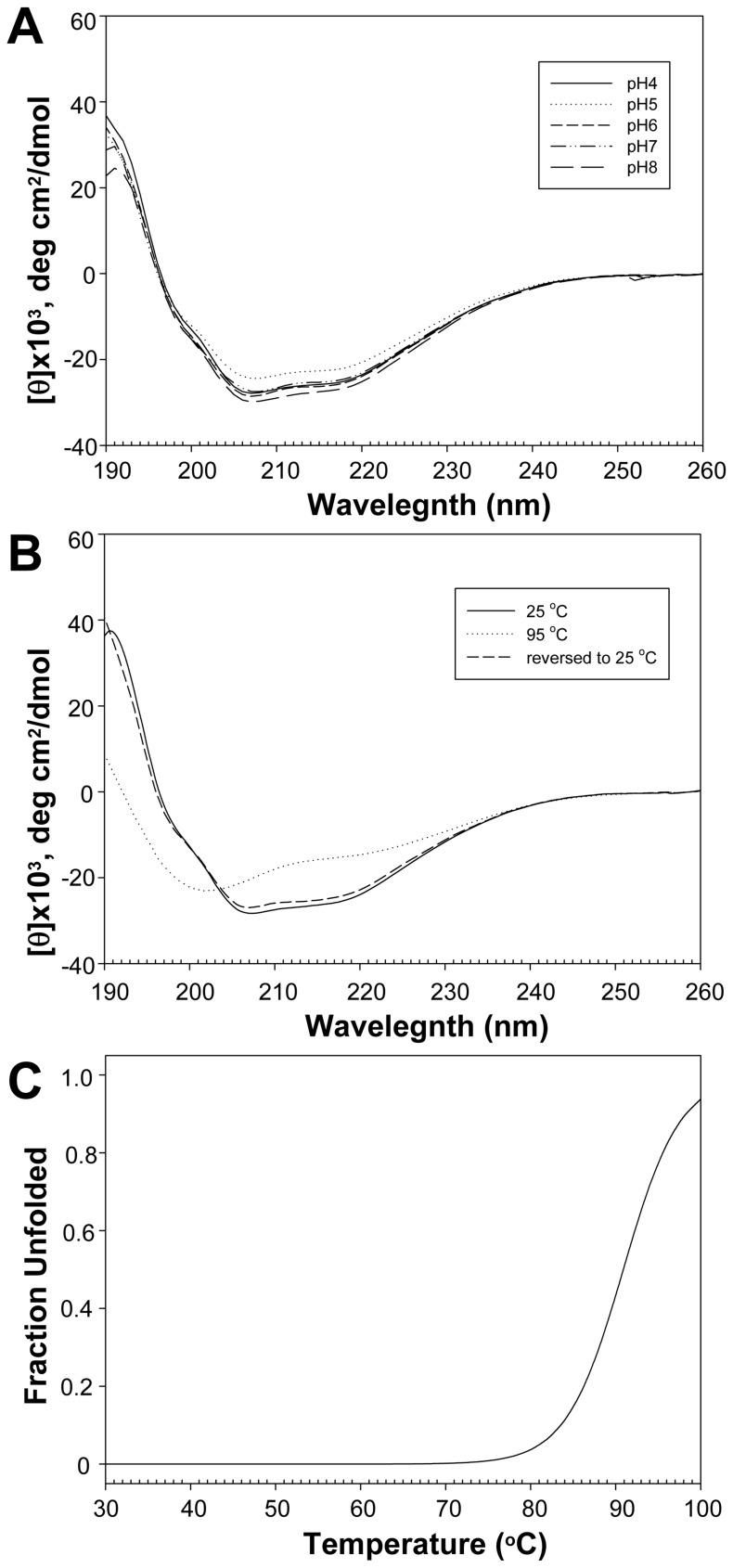
Stability of MIEN1 estimated by circular dichroism (CD) spectroscopy. (A) CD spectra of MIEN1 show a stable fold at various pH values. (B) MIEN1 protein presents a highly thermo-reversible property. (C) The Tm of MIEN1 can only be roughly estimated as more than 90°C, because an unfolded baseline cannot be established.

We constructed the full-length MIEN1 with a His-tag at the N terminus and produced it by bacterial expression as described [9]. After purification and cleavage of the His-tag with thrombin under non-denaturing conditions, we obtained MIEN1 with two extra amino acids in the N terminus. The extent of secondary structural changes in MIEN1 at different pH values and temperature was measured by far-UV CD spectroscopy. The recombinant protein MIEN1 showed a typical α/β type signal in a far-UV CD spectrum (Figure 1A, solid line). In addition, the far-UV CD spectra for MIEN1 at various pH values were almost indistinguishable, with a maximum at 190 nm and 2 minima, at 206 and 219 nm (Figure 1A). These far-UV CD data suggest that lowering the pH did not disrupt the overall secondary structure of the protein. The contents of the secondary structures of MIEN1 at different pH values were estimated by use of CONTIN-LL, SELCON3, and CDSSTR [10] (Figure S1). The mean contents of the α-helix, β-strand, turn, and coil forms are 55%, 20%, 10%, and 15%, respectively (Figure S1). The secondary structure of MIEN1 was greatly distorted on heating to 95°C monitored by a series of CD spectra at various temperatures (Figure S2). However, the CD spectrum of MIEN1 when heating was reversed to 25°C was almost identical to the original spectra at 25°C, which indicates that the thermal denaturation of MIEN1 is reversible (Figure 1B). In addition, the T*m* value was about 90°C (Figure 1C), which suggests the high thermostability of MIEN1.

### Structure Determination of MIEN1 by NMR Spectroscopy

For structure determination, MIEN1 was produced by recombinant expression in uniformly ^15^N- or ^15^N/^13^C-labeled medium. The well-dispersed signals of a ^1^H-^15^N heteronuclear single quantum coherence (HSQC) spectrum for MIEN1 suggests a folded peptide chain [9]. ^1^H, ^15^N, and ^13^C resonances for this recombinant MIEN1 protein were assigned by standard triple resonance experiments and deposited in the Biological Magnetic Resonance Bank under accession number 16413.

**Figure 2:**
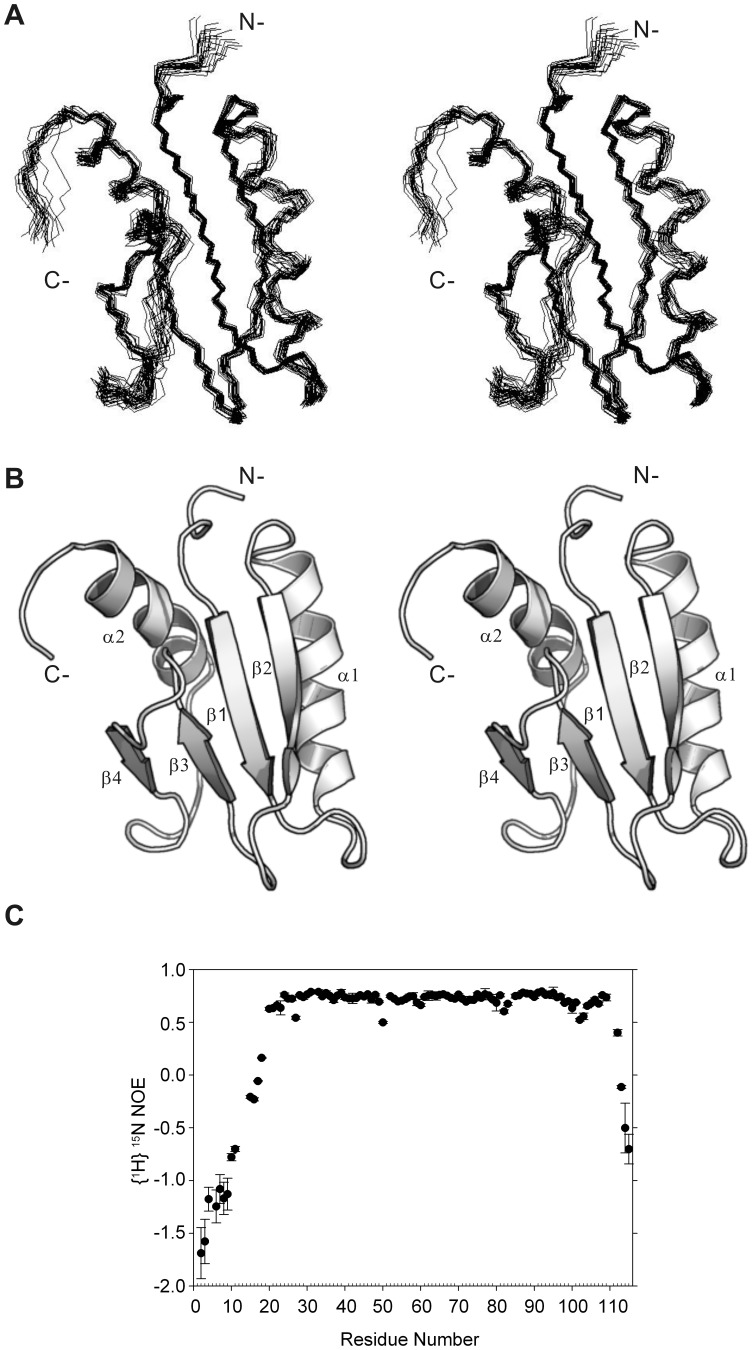
Solution structure of recombinant MIEN1. (A) Stereoviews of the final 20 MIEN1 structures are superimposed. The superimposition was optimized for backbone atoms (N, C^α^, and C’) of residues 17–102 to the mean coordinates. Residues for the nonstructured N (Met1-Glu16) and C (Glu103-Leu115) termini are not shown. (B) Stereoviews of the ribbon representation of MIEN1 protein (amino acids Val17-Leu102). (C) [^1^H-^15^N] heteronuclear NOE measured for MIEN1 protein. The intensity ratio between spectra with and without ^1^H saturation is given as a function of residue number.

We used 1,457 non-redundant and structurally restraining NOE-derived distance restraints, 8 hydrogen bond restraints, and 115 dihedral angle restraints to generate the solution structure of MIEN1 protein by the structure calculation program CYANA [11]. The energy-minimized structures with the lowest target function are in Figure 2A. The solution structure of the MIEN1 protein was well defined, with backbone root mean square deviation (RMSD) values of 0.89±0.16 Å for backbone coordinates and 1.55±0.21 Å for all heavy atoms in the structure region of residues 17–104. For secondary structure regions, the RMSD decreased to 0.68±0.16 Å and 1.41±0.18 Å for backbone and all heavy atoms, respectively. More than 99.4% of the backbone torsion angle, (φ,ψ) pairs, were in the most favored or additionally allowed regions of the Ramachandran plot (Figure S3). Structural statistics are listed in Table 1. On average, 13 NOE distance restraints per residue were used in the final structure calculations, and all NOE assignments are consistent with a monomeric structure.

**Table 1:** Characterization of input NMR data and structural statistics for the solution structure of the recombinant MIEN1 protein.

Parameters	Values
NOE distance constraints	
Total NOE	1457
Intraresidue	348
Interresidue	1109
Sequential (|i-j| = 1)	484
Medium Range (|i-j|< = 4)	306
Long Range (|i-j|> = 5)	319
Hydrogen bonds	8
Dihedral angle restraints	
PHI	57
PSI	58
**Structure statistics from CYANA, 20 conformers**	
Target function value(Å^2^)	1.54±0.25
Violation RMSD	
Average upper distance limit	0.0093
Average lower distance limit	0.0294
Average van der Waals	6
Average torsion angles	0.5507
Root mean sequence deviation to the average coordinates (Å)	
N, C^α^, C’ (residues 17–102)	0.89±0.16
Heavy atoms (residues 17–102)	1.55±0.21
N, C^α^, C’ (secondary structure)	0.68±0.16
Heavy atoms (secondary structure)	1.41±0.18
PROCHECK Ramanchandran plot analysis	
Residues in favored regions (%)	75.8
Residues in additionally allowed regions (%)	23.6
Residues in generously allowed regions (%)	0.3
Residues in disallowed regions (%)	0.3

### Overall Description of the MIEN1 Protein Structure

MIEN1 contains a central α/β core domain consisting of a mixed parallel/anti-parallel four-stranded β-sheet with two distorted α-helices (Figure 2B). Two helices are composed of residues Phe35-Gln49 (α1) and Glu86-Asn98 (α2), and the strands range from Glu54 to Leu59 (β2), Arg24 to Tyr29 (β1), Phe65 to Glu68 (β3) and Val74 to Ser76 (β4). The hydrophobic core of the protein is formed by residues Tyr39, Leu42, Val46, Tyr50, Ile53, Phe65, Ile67, Ile69, Val74, Leu89, Ile90 and Ile93. Residues in the N- and C-terminal regions (Met1-Glu16 and Glu103-Leu115, respectively) present as an unordered structure. To characterize the backbone flexibility of MIEN1, we performed ^1^H-^15^N heteronuclear NOE experiments (Figure 2C). The results matched the structural organization of this protein domain. Most of the core domain showed heteronuclear NOEs between 0.7 and 0.8, which indicates high-order parameters of a rigid protein structure, with the well-aligned β-strands showing the highest values and the loop regions showing low NOEs. The solvent-exposed N-terminal loop from 1 to 17 shows the lowest ^1^H-^15^N NOEs in accordance with the increased flexibility of this region, which suggests lack of stabilizing long-range interactions with the rest of the protein.

### Structural Similarities Search Revealed MIEN1 with a Thioredoxin-like Fold

**Figure 3:**
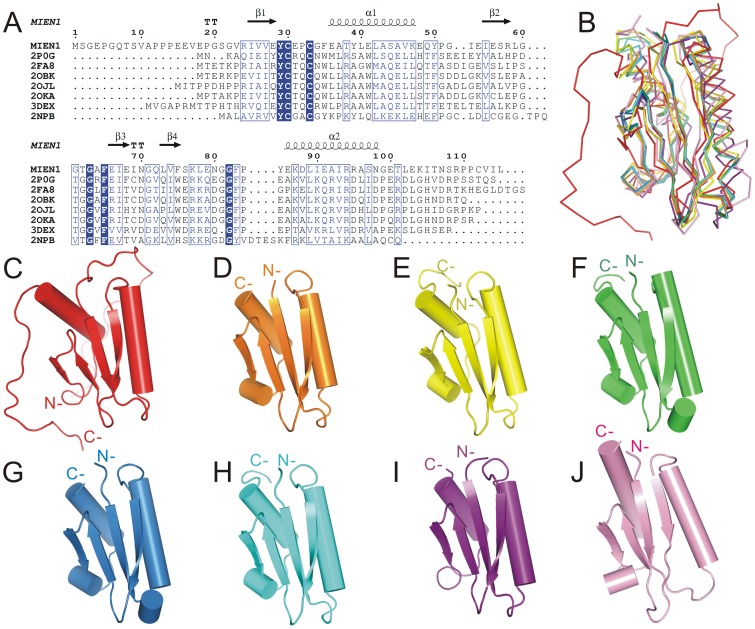
Structural comparison of MIEN1 with relatives. (A) Multiple sequence alignments based on structural comparison. Protein names, except for MIEN1, are according the PDB accession numbers: 2P0G, 2FA8, 2OBK, 2OJL, 2OKA, 3DEX and 2NPB. (B) Superposition of MIEN1 (red) and structural relatives shown as a C^α^-trace indicates the similar core domains. (C–J) Structural relatives of MIEN1. The Dali server was used to identify proteins with similar structure to MIEN1. MIEN1 (C; red), 2P0G (D; orange), 2FA8 (E; yellow), 2OBK (F; green), 2OJL (G; blue), 2OKA (H; cyan), 3DEX (I; purple) and 2NPB (J; pink).

A search of structural homology with the Dali sever identified several proteins as the closest structural relatives to MIEN1, with Z-score >7 (Table 2). The sequence identities of MIEN1 and these proteins were <24%. These related sequences are selenoprotein W-related proteins from *Vibrio cholerae* (PDB code 2P0G) and *Agrobacterium tumefaciens* (2FA8); SelT/SelW/SelH selenoprotein domain from *Pseudomonas fluorescens* (2OBK); hypothetical proteins from *Bordetella parapertussis* (2OJL), *Pseudomonas aeruginosa* (2OKA), and *Streptomyces avermitilis* (3DEX); and mouse SelW (2NPB). Six of these related proteins are bacterial proteins annotated as hypothetical or SelW-related proteins and the last one is mouse selenoprotein W [12]. Multiple sequence alignment of MIEN1 and these proteins based on structural similarity revealed a conserved CXXC motif (UXXC motif in selenoprotein) and several aromatic residues, such as Tyr29, Phe65 (Figure 3A). Although the sequence identity is low, the proteins shared a similar thioredoxin-like fold (Figure 3B). Thus, MIEN1 may belong to the thioredoxin-like protein family with its two-layer α/β sandwich of a β-α-β-β-β-α secondary-structure pattern. Structural comparison of MIEN1 and its structural relatives is in Figure 3 (C to J).

**Table 2:** DALI comparisons using the structure of MIEN1 protein that is closest to the mean of the ensemble PDB Z-score Sequence Identity Protein.

Symbol	PDB	Z-score	RMSD	Sequence Identity	Protein
A	2LJK			This study	Human MIEN1
B	2P0G	8.5	4.062	19%	Selenoprotein W-related protein from *Vibrio cholerae*
C	2FA8	8.3	2.36	18%	Selenoprotein W-related protein from *Agrobacterium tumefaciens*
D	2OBK	8.3	4.03	19%	SelT/SelW/SelH Selenoprotein domain from *Pseudomonas fluorescens*
E	2OJL	8.2	4.125	18%	Hypothetical protein from *Bordetella parapertussis*
F	2OKA	8.0	4.073	21%	Hypothetical protein from *Pseudomonas aeruginosa*
G	3DEX	8.0	2.974	16%	Hypothetical protein from *Streptomyces avermitilis*
H	2NPB	7.2	3.361	24%	mouse SelW

### Structural Effect of Active-site Disulfide Bond Reduction

**Figure 4:**
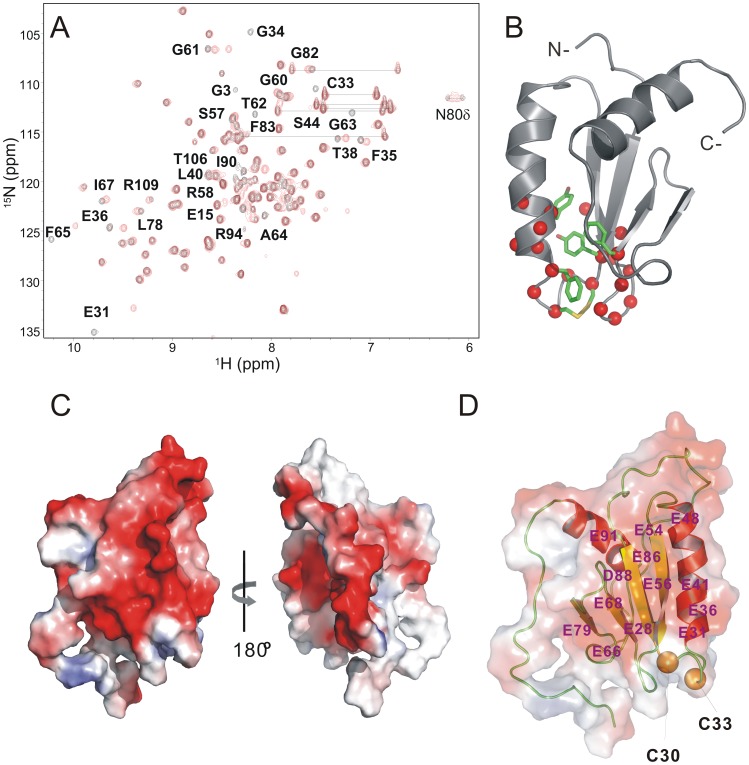
Identification of residues affected by thiol-disulfide exchange and electrostatic surface potential in MIEN1. (A) ^1^H-^15^N HSQC spectra of MIEN1 without (black) and with (red) 2 mM dithiothreitol shows chemical shift changes of several amide protons. (B) Amide protons of residues experiencing minimum chemical shift changes above the mean (Δδ > 0.11 ppm) shown as red spheres. Yellow sticks indicate the location of the cysteine residues composed of the active-site redox motif. (C) Distribution of charges on the surface of MIEN1. Red areas indicate negative charges and blue positive charges. (D) The positions of negatively charged residues, including Glu28, Glu31, Glu36, Glu41, Glu48, Glu54, Glu56, Glu66, Glu68, Glu79, Asp88, and Glu91 are indicated. The Cys30 and Cys34 residues are shown as yellow spheres.

We performed further experiments to determine the conformational differences and activities associated with the redox potential of MIEN1. A comparison of the oxidized and reduced ^1^H-^15^N HSQC spectra demonstrated that most of the backbone amide resonances were unchanged with the reduction of the active-site disulfide bond, so the thiol-disulfide exchange is not accompanied by a large conformational change. The chemical shift dispersion of the ^1^H-^15^N HSQC spectra for the oxidized and reduced forms of MIEN1 (Figure 4A) indicated that MIEN1 protein remains folded regardless of oxidation state. However, some residues showed significant changes in backbone amide resonances that are consistent with a localized structural rearrangement centered on the predicted active-site redox motifs of MIEN1 (Figure 4A and 4B).

The side chains of several aromatic residues in the MIEN1 structure form a cluster right behind the predicted active site of MIEN1 (Figure 4B). This cluster is situated too far from the CXXC motif to have a direct role in the redox reaction. However, the cluster might be relevant for binding interaction partners and/or maintaining the overall protein structure. The electrostatic potential surface of MIEN1 is shown in Figure 4C. The side of the protein predominantly shows a negative patch with some scattered neutral regions. The exclusively negative electrostatic potential surface is consistent with the total protein charge of −19 and with its estimated pI of 4.4. Of note, the charge distribution of MIEN1 differs from that of its structural relatives, which show a patch of positive charge above the CXXC (CXXU) motif (Figure S4); however, MIEN1 shows a strong negative charge for the same region. Around this area is a set of negatively charged residues, namely Glu28, Glu31, Glu36, Glu41, Glu48, Glu54, Glu56, Glu66, Glu68, Glu79, Asp88, and Glu91. Most conserved positively charged residues of the structural relatives are replaced by Thr36, Leu78, Ile90, Ala92 and Thr101 in the MIEN1 sequence. In addition, the conserved Trp residue near the active motif is replaced by a Phe residue in MIEN1. Therefore, MIEN1 may be a unique thioredoxin-like protein in selecting binding partners or substrates.

### Redox Potential of MIEN1 Protein

**Figure 5:**
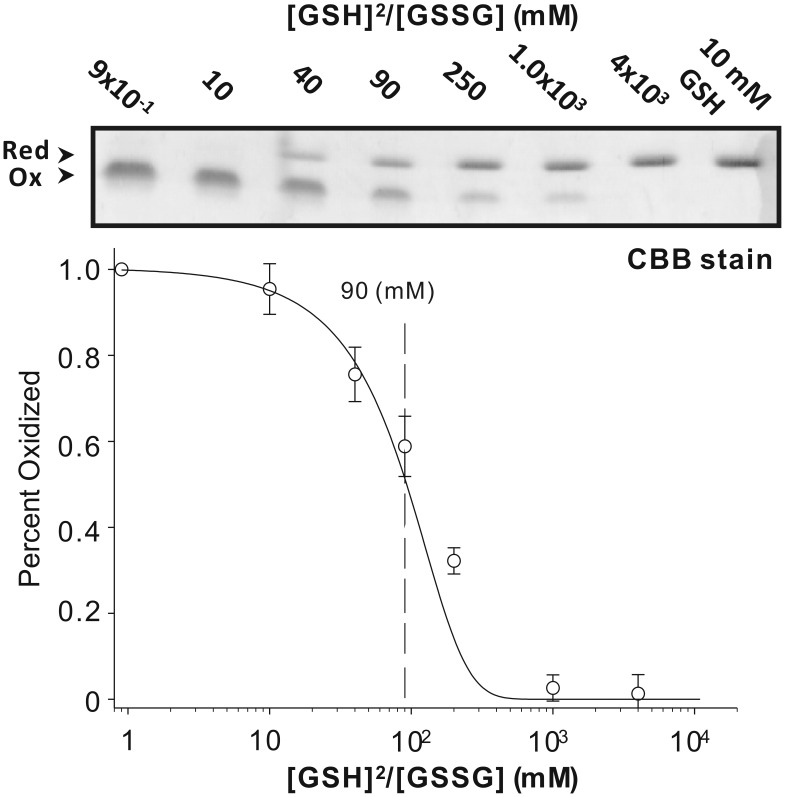
Determination of the oxidation-reduction midpoint potential of MIEN1. Redox equilibrium assay with glutathione at 25°C (top). The free sulfhydryl groups of the cysteine residues were modified with use of AMS after incubation with different [GSH]^2^/[GSSG] ratios. (Bottom) measured redox equilibrium constant of MIEN1. The apparent equilibrium constant between MIEN1 and glutathione was determined by nonlinear least square fitting of the data in the upper column.

From the structural analysis and NMR perturbation experiments, MIEN1 was found potentially involved in the formation, reduction, or isomerization of disulfide bonds. The propensity of MIEN1 to donate or accept electrons can be expressed as an equilibrium redox potential. To further characterize MIEN1, we determined the redox potential relative to that of glutathione in that reduced glutathione can transfer electrons to a protein. We determined K_eq_, 90 mM, by measuring the formation of alkylated protein with reduction at a range of ratios of [GSH]^2^/[GSSG] (Figure 5). The redox potential of −225 mV was further calculated by use of the Nernst equation. Intriguingly, the calculated redox potential of MIEN1 was higher than that of the strong disulfide reductant thioredoxin (−270 mV) and lower than that of the disulfide oxidase DsbA (−122 mV). The redox potential of MIEN1 was found between that of thioredoxin and protein disulfide isomerase (−175 mV), so MIEN1 may catalyze the reduction and/or isomerization of disulfide bonds for tumor-related proteins. The redox potential of MIEN1 compares well with that of thioredoxin-related proteins, ranging from −147 to −270 mV [13].

## Discussion

To establish the oncogenic-potential MIEN1 protein as a therapeutic target for cancer treatment, the most important task is to detail its functional significance. Solving the MIEN1 structure allowed for interpreting the sequences of homologous proteins and predicting their structures. Here we describe the solution structure of MIEN1 protein by high-resolution NMR spectroscopy. The structure revealed a thioredoxin-like fold, a β-α-β-β-β-α secondary structure core, and a CXXC motif in an exposed loop in a position similar to the redox-active CXXC site in thioredoxin. Analysis of protein dynamics revealed rigidity in the backbone, except for the 20 residues at the N terminus and 5 residues at the C terminus. The NMR-derived structure also suggested a role for MIEN1 in redox regulation. The observed localized conformational changes after thiol-disulfide exchange and measurement of redox potential indicate that MIEN1 has redox activity.

Many cellular processes are regulated by reduction and oxidation (redox) by a set of proteins. The redox function of these proteins may be supported by protein-bound cofactors or involve redox-active cysteine residues [14]. Previous study of thiol oxidoreductases has focused on proteins containing the thioredoxin fold that are involved in regulating intracellular redox homeostasis and redox-sensitive protein-folding pathways [15]. However, these proteins are widespread, and most have not been functionally characterized [16].

The classical thioredoxin fold is described as a three-layer α/β/α sandwich composed of a mixed five-stranded β-sheet and 2 pairs of α-helices on either side of the β-sheet [17]. Recent comprehensive structural classification described the thioredoxin-like fold as a two-layer α/β sandwich composed of a mixed four-stranded β-sheet and a pair of α-helices packed against one side of the β-sheet [18], [19]. Although MIEN1 shares low sequence identity with other proteins containing thioredoxin-like domains, the NMR structure revealed the most basic thioredoxin-like fold, which implies that MIEN1 belongs to the thioredoxin-like protein family. In addition, the thioredoxin-like domain of MIEN1 shows that the molecule has a very tight fold, with >60% of its primary sequence involved in strong elements of secondary structure and high-order NMR dynamics parameters. This provides an explanation for MIEN1’s observed high thermal stability, and suggests that the thioredoxin-like protein family might possess the robust folding characteristics.

**Figure 6:**
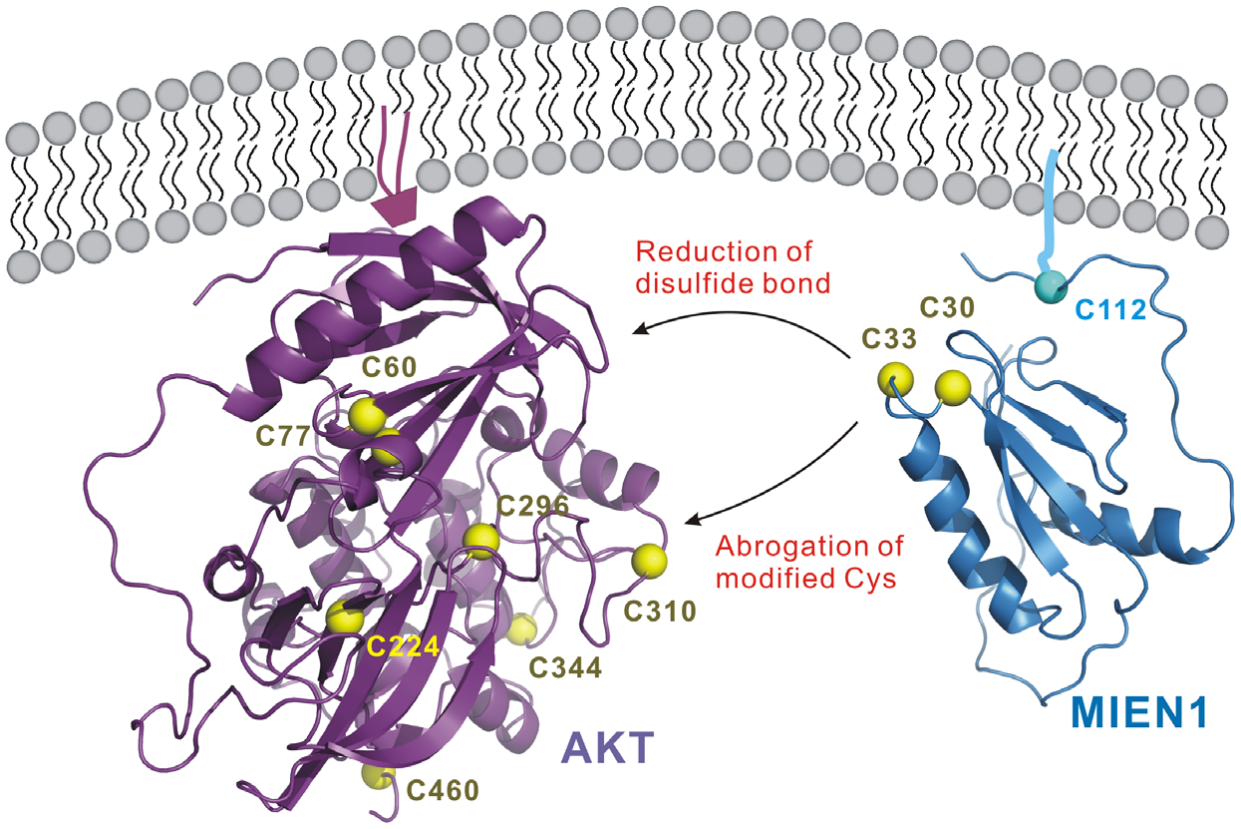
Proposed redox-regulated mechanism of AKT activation by MIEN1. Signal activating AKT translocates to the membrane. Prenylated protein MIEN1 also remains localized to the cytosolic face of the membrane and in turn controls the downstream signaling of phosphorylated AKT by changing the disulfide bond or modified cysteine on AKT for following activation. The cysteine residue is represented as a sphere.

Previous reports showed that MIEN1 expression is highly related clinically to breast [1] and prostate cancer [3]. Overexpression of MIEN1 could promote cell migration and invasion via increasing amount of phosphorylated AKT [3]. However, the functional significance of MIEN1 in cancer biology remained largely unknown. Here, we first proposed molecular function of MIEN1 may act as a redox regulator in cellular signaling, based on the determined thioredoxin-like fold and redox potential evidence. The redox-regulated switch has been shown as an important regulatory system in many signal transduction pathways [20], [21], [22]. Intriguingly, activity of AKT, also located on the cystosolic face of the membrane, was reduced with *MIEN1* gene silencing (Figure 6), which indicates that membrane-bound prenylated MIEN1 may be a potential regulator of AKT [3]. From our protein dynamic experiments of MIEN1, the C-terminal tail region (98–110) before the prenylation site shows an unexpected behavior in that its flexibility seems not to be as high as in the N terminus (Figure 2C). Interestingly, the extra turn structure (98–102) after the thioredoxin-like domain seems to orient the redox-active motif closer to the membrane side. Thus, the relatively rigid conformation of the extra turn should be part of the functional thioredoxin-like domain of MIEN1 and direct prenylated MIEN1 to regulate membrane-bound signaling proteins such as AKT. Full activation of AKT is a multi-step process, and several proteins responsible for each step have been identified and characterized [23], [24], [25]. On stimulation, phosphorylation of the activation loop (A-loop) and hydrophobic motif (HM) of AKT is by PDK1 and mTORC2, respectively. A well-conserved threonine in the turn motif (TM) is also constitutively phosphorylated by mTORC2 and contributes to the stability of AKT [26], [27], [28]. Of note, the inactive unphosphorylated AKT protein structure suggests that inactive conformations are contributed by an intra-molecular disulfide bond in the active site, as well as residues in a region between the N- and C- terminal lobes [26], [27], [29]. In addition, nitric oxide donors were found to inactivate AKT both *in vitro* and in intact cells with simultaneous S-nitrosylation at Cys224 of AKT in insulin resistance [30]. Therefore, MIEN1 may contribute to the reduction of intra-molecular disulfide bond between Cys297 and Cys311 in the A-loop and the modification of Cys224 or Cys460 around the TM and HM of inactivated AKT, thus allowing for conformational change for phosphorylation (Figure 6). This possibility deserves further investigation.

## Materials and Methods

### Production of Recombinant MIEN1 Protein

The expression plasmids encoding full-length MIEN1 cDNA were generated by one-step RT-PCR of breast cancer cells, SK-BR-3 (obtained from ATCC), with the primer, forward 5′-ACGGATCCGCGATGAGCGGGGAGCC-3′ containing a *Bam*HI restriction site, and reverse, 5′-GCGAATTCGGCTGGAGAAGTGTTCATGGAG-3′ containing an *Eco*RI restriction site. Production and purification of recombinant MIEN1 protein with His-tag followed published protocols [9]. The uniformed ^15^N or ^13^C-labeled protein was produced in *Escherichia coli* BL21 (DE3) from the *MIEN1* gene cloned in pET-28a (Novagen), induced by IPTG and purified by use of the Ni-NTA column (GE, Healthcare). Recombinant protein was then digested with thrombin to remove the His-tag and purified by use of the size-exclusion Superdex75 XK 16/60 column (GE, Healthcare). Finally, MIEN1 protein (13 kDa, with an extra Gly and Ser at the N terminus) was concentrated to 1 mM in 20 mM sodium phosphate (pH 6.5) and 100 mM NaCl.

### CD Spectroscopy

For CD experiments, 400 µl protein samples of 30 µM were placed into a 1-mm pathlength cuvette and maintained at 25°C. The change in far-UV CD spectra from 190 to 260 nm at various pH values was measured by use of a JASCO J-810 spectropolarimeter equipped with a Peltier temperature control system (Japan Spectroscopic Co., Tokyo). After background subtraction and smoothing, all CD data were converted from CD signals (millidegree) to mean residue ellipticity (deg cm^2^ dmol^-1^). The CD spectra were analyzed by use of CDPro [10]. Thermal transition of 30 µM MIEN1 protein in PBS (pH 7.0) was monitored at 218 nm from 10 to 96°C with a scan rate of 2 deg C/min. The equilibrium denaturation curve was fitted to a two-state model as previously described [31] and the observable ellipticity values [*θ*]_obs_ was calculated as [*θ*]_obs_ = [*θ*]_N_ *f*_N_+[*θ*]_U_ *f*_U_, where [*θ*]_N_ and [*θ*]_U_ are the pre- and post-transition temperature-dependent baselines, respectively. *f*_N_+*f*_U_ = 1, where *f*_N_ and *f*_U_ represent the fraction protein present in the native and unfolded conformations, respectively. The alternative equation can be presented as *f*_U_ = ([*θ*]_N_-[*θ*]_obs_)/([*θ*]_N_−[*θ*]_U_).

### NMR Experiments

The NMR spectra were acquired on Bruker AV600 and AV800 spectrometers equipped with 5-mm triple resonance cryoprobe and single-axis pulsed-field gradient at 310 K as described [9], [32], [33]. NMR data were collected in Shigemi tubes with 1-mM protein samples in 20 mM Na_3_PO_4_, 50 mM NaCl, 10% D_2_O at pH 6.5. Protein backbone resonance assignments were based on standard triple resonance experiments [34], [35], [36], [37]: HNCA, HNCACB, CBCA(CO)NH, HNCO and HN(CA)CO. Aliphatic side-chain assignments primarily involved HCCH-TOCSY and HCCH-COSY with the help of HCC(CO)NH and HBHA(CO)NH experiments [38]. ^15^N-edited TOCSY-HSQC acquired with 60-ms mixing time was used to resolve ambiguity due to spectral overlap. Aromatic side chains were assigned with ^13^C-edited NOESY-HSQC from aliphatic and aromatic region experiments [39]. The data were acquired and processed by use of Topspin2.1 (Bruker, Germany) and further analyzed by use of Sparky 3.114 (Goddard and Kneller). ^1^H chemical shifts were externally referenced to 0 ppm of 2,2-dimethyl-2-silapentane-5-sulfonate, whereas ^13^C and ^15^N chemical shifts were indirectly referenced according to IUPAC recommendations [40]. The steady-state heteronuclear [^1^H-^15^N] NOE experiment was carried out in duplicate in an interleaved manner, with and without proton saturation. The NOE was calculated as the error-weighted average ratio of peak intensities, with error estimate by propagating the base-plane noise. Chemical shift differences between the backbone amide ^1^H and ^15^N resonances of oxidized versus reduced MIEN1 were calculated by the equation: Δδ  =  ([Δδ^1^H]^2^ + 0.1 × [Δδ^15^N]^2^)^1/2^ [41].

### Structure Calculation

The sequence, chemical shift, and cross-peak files were converted to XEASY format by use of the CCPN format converter [42]. CYANA [11] was used to compute 6 cycles, each with 600 structures. Backbone torsion angle restraints of PHI and PSI in the regular secondary structure regions were derived by use of TALOS [43] and used during the CYANA calculation. Input data and structure calculation statistics are listed in Table 1. The quality of the structure was evaluated by use of PROCHECK-NMR [44]. Structures were analyzed and represented by use of MOLMOL [45] and PyMol [46].

### Redox Potential Assay

The redox equilibrium between recombinant MIEN1 and glutathione was measured as follows. MIEN1 protein (1 µM) was incubated with 0.1 mM GSSG and concentrations of GSH at 25°C for 1 h in 0.1 M phosphate buffered saline (pH 7.0) containing 1 mM EDTA. After incubation, trichloroacetic acid (10%) was added to prevent further thiol-disulfide exchange. The precipitated pellet was solubilized in 0.1 M phosphate buffered saline (pH 7.0) containing 2% SDS and 1 mM 4-acetamido-4′-maleidylstilbene-2,2′-disulfonic acid (AMS; Invitrogen), then incubated at 25°C for 1 h to alkylate free sulfhydryl groups of cysteines. The samples were separated by 12.5% SDS-PAGE and stained with Coomassie Brilliant Blue [47]. The ratio of oxidized form was quantified and compared the intensities of bands on the gel using ImageJ [48]. The redox equilibrium constant (*K*_eq_) was calculated by fitting the fraction of the reduced form to the following equation: *r = *([GSH]^2^/[GSSG])/(*K*eq+([GSH]^2^/[GSSG])) [49], where *r* is the relative ratio of reduced MIEN1.

## Supporting Information

Figure S1 The contents of the secondary structures of MIEN1 in different pH values were estimated using CONTIN-LL, SELCON3, and CDSSTR. The average contents of the α-helix, β-strand, turn, and coil forms are around 55, 20, 10, and 15%, respectively.
(TIF)

Figure S2 CD spectra of MIEN1 were collected at various temperatures (20, 30, 40, 50, 60, 70, 80, and 90°C).
(TIF)

Figure S3 Ramachandran plot for the phi-psi values of the final 20 structures of recombinant MIEN1. This figure was produced using PROCHECK-NMR.
(TIF)

Figure S4 Surface electrostatic potential comparison of MIEN1 with structural relatives. Two different orientations of the protein surface electrostatic potential related by 180° rotation along the *vertical axis*. Positive (blue) and negative (red) electrostatic potentials of each molecule are mapped on the van der Waals surfaces. The protein names are as follows: MIEN1(A), 2P0G(B), 2FA8(C), 2OBK(D), 2OJL(E), 2OKA(F), 3DEX(G) and 2NPB(H).
(TIF)
