# Supplementary figures and images for: Solution Structure of the Oncogenic MIEN1 Protein Reveals a Thioredoxin-Like Fold with a Redox-Active Motif
Source: PLoS One. 2012 Dec 20;7(12):e52292. doi: 10.1371/journal.pone.0052292 (PMC3527542; doi:10.1371/journal.pone.0052292)

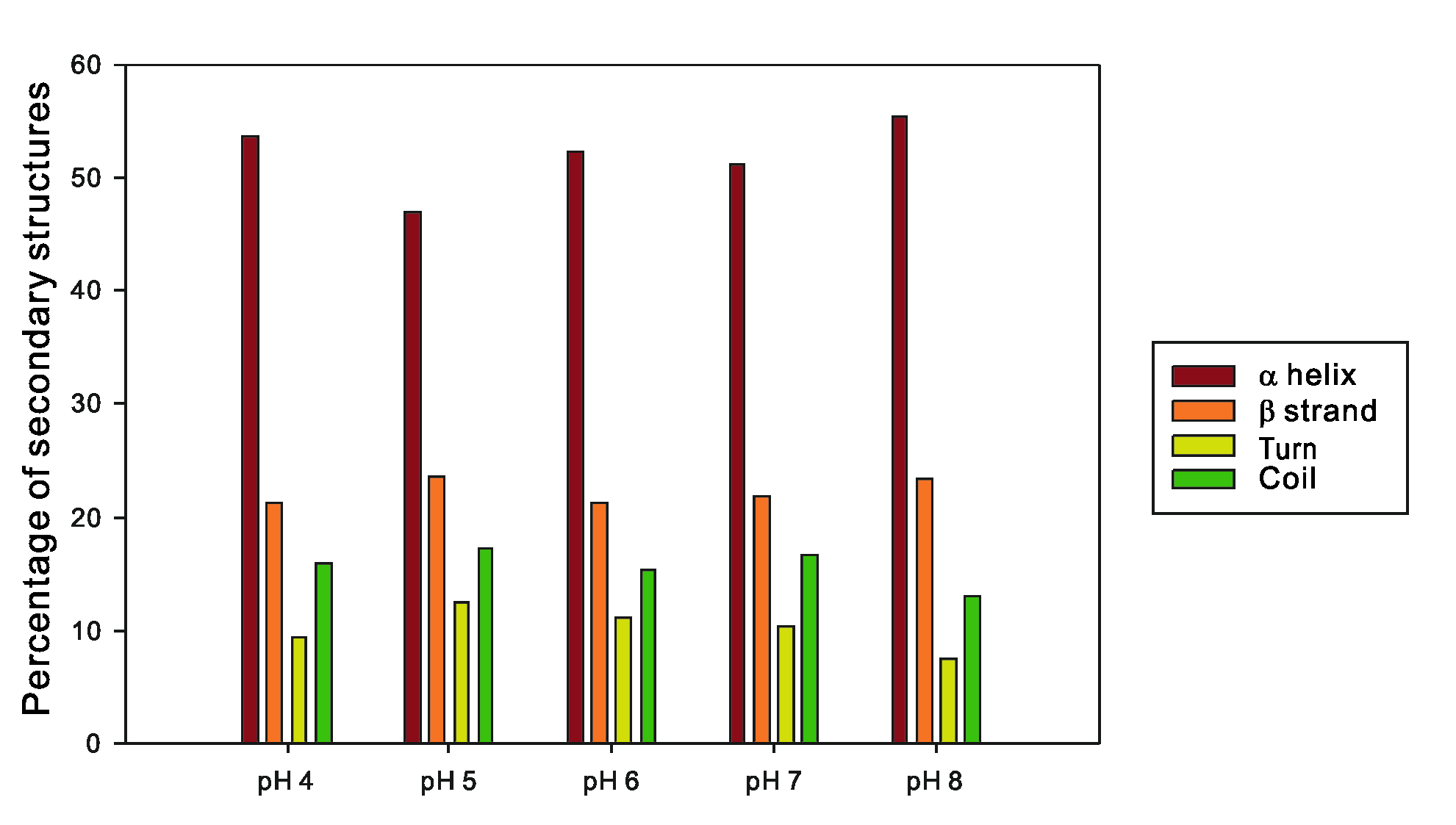

Supplement: Figure S1 — The contents of the secondary structures of MIEN1 in different pH values were estimated using CONTIN-LL, SELCON3, and CDSSTR. The average contents of the α-helix, β-strand, turn, and coil forms are around 55, 20, 10, and 15%, respectively. (TIF) [file pone.0052292.s001.tif]

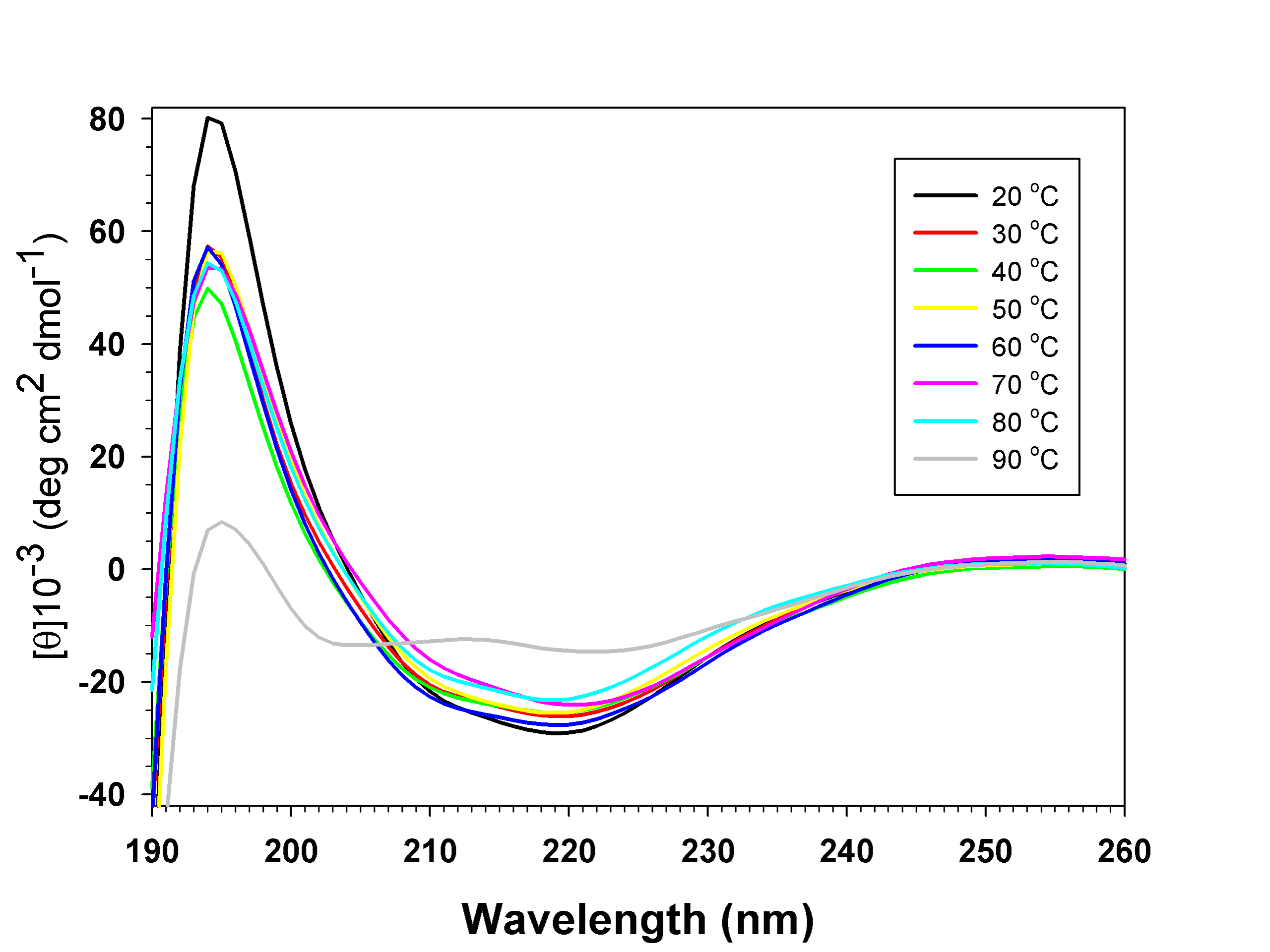

Supplement: Figure S2 — CD spectra of MIEN1 were collected at various temperatures (20, 30, 40, 50, 60, 70, 80, and 90°C). (TIF) [file pone.0052292.s002.tif]

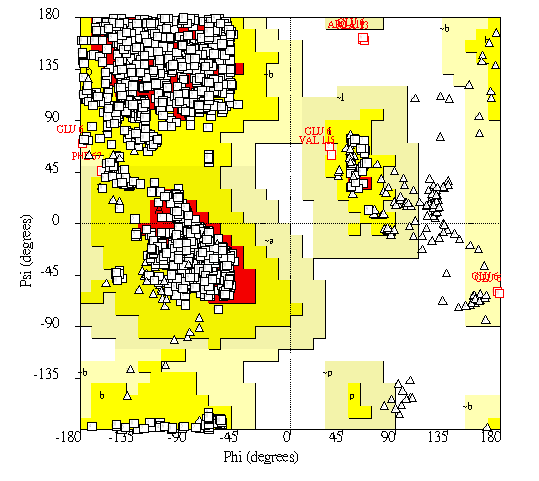

Supplement: Figure S3 — Ramachandran plot for the phi-psi values of the final 20 structures of recombinant MIEN1. This figure was produced using PROCHECK-NMR. (TIF) [file pone.0052292.s003.tif]

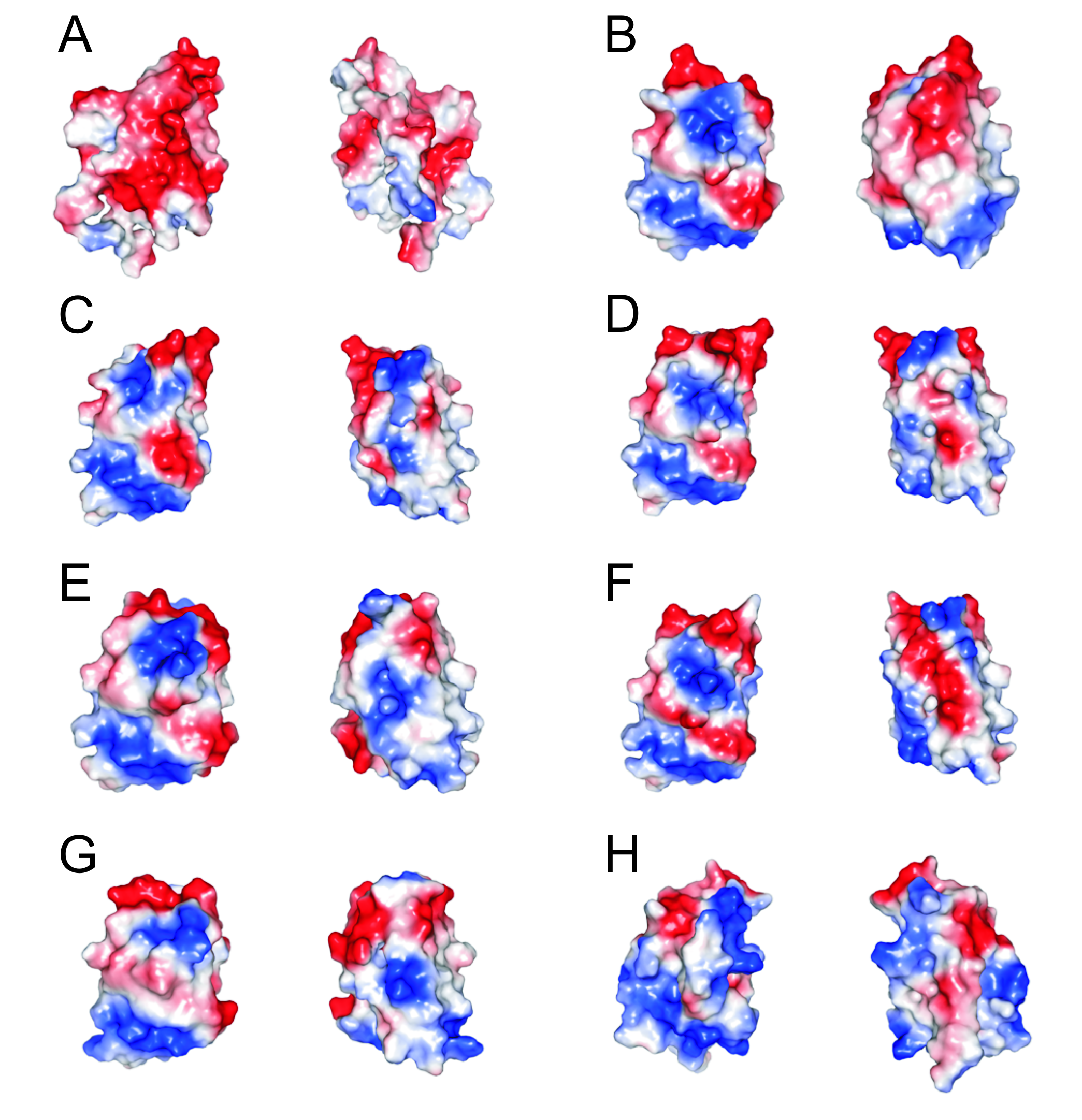

Supplement: Figure S4 — Surface electrostatic potential comparison of MIEN1 with structural relatives. Two different orientations of the protein surface electrostatic potential related by 180° rotation along the vertical axis. Positive (blue) and negative (red) electrostatic potentials of each molecule are mapped on the van der Waals surfaces. The protein names are as follows: MIEN1(A), 2P0G(B), 2FA8(C), 2OBK(D), 2OJL(E), 2OKA(F), 3DEX(G) and 2NPB(H). (TIF) [file pone.0052292.s004.tif]
